# Carbapenem resistance gene crisis in *A. baumannii:* a computational analysis

**DOI:** 10.1186/s12866-022-02706-8

**Published:** 2022-12-03

**Authors:** Nureen Zahra, Basit Zeshan, Musarat Ishaq

**Affiliations:** 1 Department of Microbiology, Faculty of Science & Technology, University of Central Punjab, Lahore, Pakistan; 2 Institute of Molecular Biology and Biotechnology, The University of Lahore, Lahore, Pakistan; 3 Faculty of Sustainable Agriculture, Universiti Malaysia Sabah, 90905 Sandakan, Sabah Malaysia; 4 Lymphatics and Regenerative Surgery Laboratory, Obrien Institute and St Vincent’s Institute, Fitzroy, Australia

**Keywords:** Antimicrobial resistance (AMR), Computational analysis (CA), Drug design (DD), Kilocalories per mole(Kcal/mol)

## Abstract

*Acinetobacter baumannii (A. baumannii)* is one of the members of *ESKAPE* bacteria which is considered multidrug resistant globally. The objective of this study is to determine the protein docking of different antibiotic resistance gene (ARGs) in *A. baumannii*. In silico analysis of antibiotic resistance genes against carbapenem are the blaOXA-51, blaOXA-23, blaOXA-58, blaOXA-24, blaOXA-143, NMD-1 and IMP-1 in *A. baumannii*. The doripenem, imipenem and meropenem were docked to blaOXA-51 and blaOXA-23 using PyRx. The top docking energy was -5.5 kcal/mol by imipenem and doripenem and meropenem showed a binding score of -5. 2 kcal/mol each and blaOXA-23 energy was -4.3 kcal/mol by imipenem and meropenem showed a binding score of -2.3 kcal/mol, while doripenem showed the binding score of -3.4 kcal/mol. Similarly, doripenem imipenem and meropenem were docked to blaOXA-58, IMP-1, Rec A and blaOXA-143, with docking energy was -8.8 kcal/mol by doripenem and meropenem each while imipenem showed a binding score of -4.2 kcal/mol and with IMP-1 demonstrated their binding energies. was -5.7 kcal/mol by meropenem and doripenem showed a binding score of -5.3 kcal/mol, while imipenem showed a binding score of -4.5 kcal/mol. And docking energy was -4.9 kcal/mol by imipenem and meropenem showed binding energy of -3.6 kcal/mol each while doripenem showed a binding score of -3.9 kcal/mol in RecA and with blaOXA-143 docking energy was -3.0 kcal/mol by imipenem and meropenem showed a binding score of -1.9 kcal/mol, while doripenem showed the binding score of -2.5 kcal/mol respectively. Doripenem, imipenem, and meropenem docking findings with blaOXA-24 confirmed their binding energies. Doripenem had the highest docking energy of -5.5 kcal/mol, meropenem had a binding score of -4.0 kcal/mol, and imipenem had a binding score of -3.9 kcal/mol. PyRx was used to dock the doripenem, imipenem, and meropenem to NMD-1. Docking energies for doripenem were all – 4.0 kcal/mol, whereas meropenem had docking energy of -3.3 kcal/mol and imipenem was -1.50 kcal/mol. To the best of our knowledge the underlying mechanism of phenotypic with genotypic resistance molecular docking regarding carbapenem resistance *A. baumannii* is unclear. Our molecular docking finds the possible protein targeting mechanism for carbapenem-resistant *A.baumannii*.

## Introduction

According to the World Health Organization, (WHO), *A. baumannii* is one of the most important nosocomial pathogens and a great resistance model to antimicrobials [1]. Antibiotic resistance causes major problems for physicians treating infectious diseases [2]. The carbapenem drug was used as a choice of drug but with time, it’s become resistant to multidrug-resistant (MDR), pan drug-resistant (PDR) and extra drug-resistant (XDR). Major health concerns are to control the elevated resistance as transferred in the gene as antibiotic resistance gene (ARGs) [3–5].

Molecular docking provides successful insights into the structure–activity relationships, mode of activity, and further analysis from protein–ligand interaction. Such studies would culminate in the development of novel drug molecules at a faster pace against infectious pathogens. Additionally, the physicochemical properties of the molecule would provide vital information on the initial phase of drug development [6–8].

Pathogen like *A. baumannii* has a high level of resistance against available antibiotics carbapenem and colistin which are the last line of treatment [9]. Carbapenemase in carbapenem-resistant *A. baumannii* (CRAB) is the major mechanism of carbapenem resistance in *A. baumannii* as well as other Gram-negative bacteria. The carbapenemase enzyme-encoded genes are located on mobile genetic elements like plasmid, transposon and integron [10, 11]. Due to mobile genetic elements (MGEs) many carbapenem genes are transferred from plasmid to chromosome and also transferred from bacteria to bacteria by horizontal gene transfer. It has been used to characterize and understand the mechanisms of AMR and spread through bacterial species, which is necessary for combating AMR bacteria [12]. We have reported the usage of antibiotics by covid-19 patients with co-morbidity has increased the risk of antimicrobial resistance [13]. The computational analyses of the resistant gene in *A. baumannii* are important to implement the new drug for overcoming the resistance in such types of pathogens [14].

## Method

### Server and tools

All lists of electronic tools were briefly elaborated in this work as swiss prot and uniprot model.

### Preparation of target proteins

The three-dimensional (3D) structure of resistant beta-lactam oxacillinase-51, beta-lactam oxacillinase, beta-lactam oxacillinase-58, beta lactam oxacillinase-24, beta lactam oxacillinase-143, and New Delhi metallo-ß-lactamase-1) were accessed from the protein data bank (PDB) (https://www.rcsb.org/) while IMP-1 and rec A against carbapenem (Imipenem, Meropenem and doripenem) were accessed from UniProtKB/Swiss-Prot database (https://swissmodel.expasy.org/interactive) because protein structures of IMP-1 and rec A were not present in PDB.

### Homology modelling, modelling refinement and energy minimization

These proteins are involved in different activities such as antibiotic-resistant gene blaOXA-51, blaOXA-23, blaOXA-58, blaOXA-24, blaOXA-143, and NMD-1, with target antibiotics retrieved from the protein data bank (https://www.rcsb.org/) with sequence entries 5KZH (https://www.rcsb.org/structure/5KZH), 4JF5 (https://www.rcsb.org/structure/4jf5), 4OH0 (https://www.rcsb.org/structure/4oh0), 3G4P (https://www.rcsb.org/structure/3g4p), 6NZ8 (https://www.rcsb.org/structure/6nz8) 6O3R (https://www.rcsb.org/structure/6o3r) respectively and IMP-1 and rec A were retrieved from the swiss model (https://swissmodel.expasy.org/interactive) with sequence entries Q6ZXZ6 (https://www.uniprot.org/uniprotkb/Q6ZXZ6) and PS50163 (https://prosite.expasy.org/PS50163) respectively. The UCSF chimera 1.6 was used to minimise the selected protein models [15] and saved in PDB format through PyMoL [16]. Furthermore, VADAR 1.8 online server was used to calculate the protein architecture and their statistical percentage of α-helices and β-sheets, turns and coli (http://redpoll.pharmacy.ualberta.ca/vadar) and WinCoot was used to assess the Ramachandran plot value [17].

To perform structure refinement and energy minimization of the three-dimensional modelled protein structures, the online server GalaxyRefine (http://galaxy.seoklab.org/refine and YASARA software (http://www.yasara.org/) were used, respectively. GalaxyRefine employs the CASP10 assessment to refine the query structure, improving the structural and global quality of the three-dimensional model. This method initially rebuilds side chains and performs side-chain repacking and subsequently uses MD simulation to achieve overall structure relaxation.

### Selection and preparation of carbapenem

A comprehensive literature survey was employed and checked all Doripenem, imipenem and meropenem target of a particular resistance protein. Doripenem, imipenem and meropenem were retrieved from the PubChem database (https://pubchem.ncbi.nlm.nih.gov/) having accession No 73303, 104,838, 441,130 respectively and downloaded in SDF format. The retrieved antibiotics were designed in ACD/ChemSketch (https://www.acdlabs.com/) and accessed in PDB format for further docking process.

### Prediction of ligand binding site of target proteins

The active sites gene blaOXA-51, bla23, blaOXA-58, blaOXA-24, blaOXA-143, NMD-1, IMP-1 and rec A provide significant information regarding the functionality of mediated signalling pathways. The active binding sites of blaOXA-51, blaOXA-23, blaOXA-58, blaOXA-24, blaOXA-143, NMD-1, IMP-1 and rec A were predicted by using Depth Residue (http://cospi.iiserpune.ac.in/depth/), an online source which explores the probability of amino acids involved in the formation of active binding sites.

### Receptor grid generation and molecular docking

The selected ligands have their particular protein binding site; therefore, grid generation ian s important step before going to perform a docking experiment. A cubic gird box with the x-axis, y-axis and z-axis values was fixed by entering a particular size and numbers an active site in all eight selected proteins separately. The grid box of blaOXA-51 with ligands was adjusted as X = 10.5 Å, Y = 19. 1 Å and Z = 43.7 Å, similarly for blaOXA-23 the binding pocket dimensions were determined by keeping the grid size of centre X = -20.9 Å, Y = -26.3 Å and Z = 9.38 Å and Grid center of blaOXA-58 was designated at dimensions X = -16.9 Å, Y = -1.5 Å and Z = 4.15 Å. Whereas Grid box of blaOXA-24 with ligands was set as X = 88.2 Å, Y = 26.1 Å and Z = 25.8 Å, for blaOXA-143 the binding pocket dimensions were adjusted by keeping the grid size of center X = 52.8 Å, Y = 115.9 Å and Z = 22.5 Å and NMD-1 grid centre was consigned at dimensions X = 8.2 Å, Y = -3.1 Å and Z = 1.5 respectively. Similarly, for IMP-1 the binding pocket dimensions were determined by keeping the grid size of center X = -7.4 Å, Y = 1.3 Å and Z = 12.9 Å and the Grid center of rec A was adjusted at dimensions X = 11.4 Å, Y = 14.1 Å and Z = 3.9 Å respectively. Moreover, the exhaustiveness value was fixed for all docking complexes to obtain the finest binding conformational pose of selected compounds. After ligands and proteins preparation, docking was done by using PyRx a virtual screen tool [18]. The different docking complexes were analyzed based on binding energy values (Kcal/mol) and interactive behaviour such as hydrogen and hydrophobic interactions. The graphical representation of docking complexes was generated using Discovery Studio and Chimera tools, respectively.

## Results and discussion

The phenotypic and genotypic evaluation of antibiotic resistance of *A. baumannii* isolated from intensive care unit patients were reported [19, 20]. Herein, in silico studies on eight resistant genes were subjected to molecular docking against the carbapenem protein. The tested drugs exhibited a variable degree of affinity toward the resistance gene. Selim et al. 2022 [21] have conducted in silico study on the dynamics of carbapenemas*e OXA* genes towards only imipenem. The actual binding depiction of selected compounds along with the standard also represented good conformational behaviour within the active region of target proteins such as blaOXA-51, blaOXA-23, blaOXA-58, blaOXA-24, blaOXA-143, NMD-1, IMP-1 and rec A, respectively.

### Proteins structure assessment

The blaOXA-51 belongs to the hydrolase family, and comprises four chains (A, B, C and D) and 250 amino acids having a molecular mass of 113.28 kDa. The VADAR 1.8 structure analysis of blaOXA-51 consists of f 40% α-helices, 25% β-sheets, 34% coils and 5% turns, respectively. The Ramachandran plots and values of blaOXA-51 indicated that 98.94% of blaOXA-51 amino acids were present in the preferred region and 1.06% residues were in the allowed region. blaOXA-23 is another target protein consisting of a single chain (A) having 243 amino acids with a molecular weight of 27.90 kDa. The structural analysis revealed that blaOXA-23 is composed of 39% α-helices, 23% β-sheets, 36% coils and 23% turns. The Ramachandran plots and values of blaOXA-23 indicated that 98.33% of protein amino acids were present in the preferred region and 1.67% of residues lie in the allowed region. blaOXa A-58 has a single chain (A) having 280 amino acids with a molecular weight of 98.01 kDa. TRPV4 consists of 37% α-helices, 25% β-sheets, 37% coils and 21% turns, respectively. The Ramachandran plots and values of blaOXA-58 indicated that 97.90% of blaOXA-58 residues were present in the preferred region and 2.10% of residues lie in the allowed region.

The blaOXA-24 from the hydrolase family, comprises a single chain (A) having a length of 250 amino acids having a molecular mass of 27.59 kDa. The VADAR 1.8 structure analysis of blaOXA-24 consists of f 39% α-helices, 26% β-sheets, 34% coils and 5% turns, respectively. The Ramachandran plots and values of blaOXA-24 indicated that 93.80% of blaOXA-24 amino acids were present in preferred region and 4.55% residues were in allowed region. blaOXA-143 is another target protein that consists of a single chain (A) having 258 amino acids with molecular weight of 29.60 kDa. The structural analysis revealed that blaOXA-143 is composed of 38% α-helices, 25% β-sheets, 35% coils and 18% turns. The Ramachandran plots and values of blaOXA-143 indicated that 97.30% of protein amino acids were present in preferred region and 2.70% residues were lie in allowed region. NMD-1 has a single chain (A) having 233 amino acids with molecular weight of 25.02 kDa. NMD-1 consists of 25% α-helice, 31% β-sheets, 42% coils and 31% turns, respectively. The Ramachandran plots and values of NMD-1 indicated that 97.36% of NMD-1 residues were present in preferred region and 1.76% residues were lie in allowed region.

**Fig. 1:**
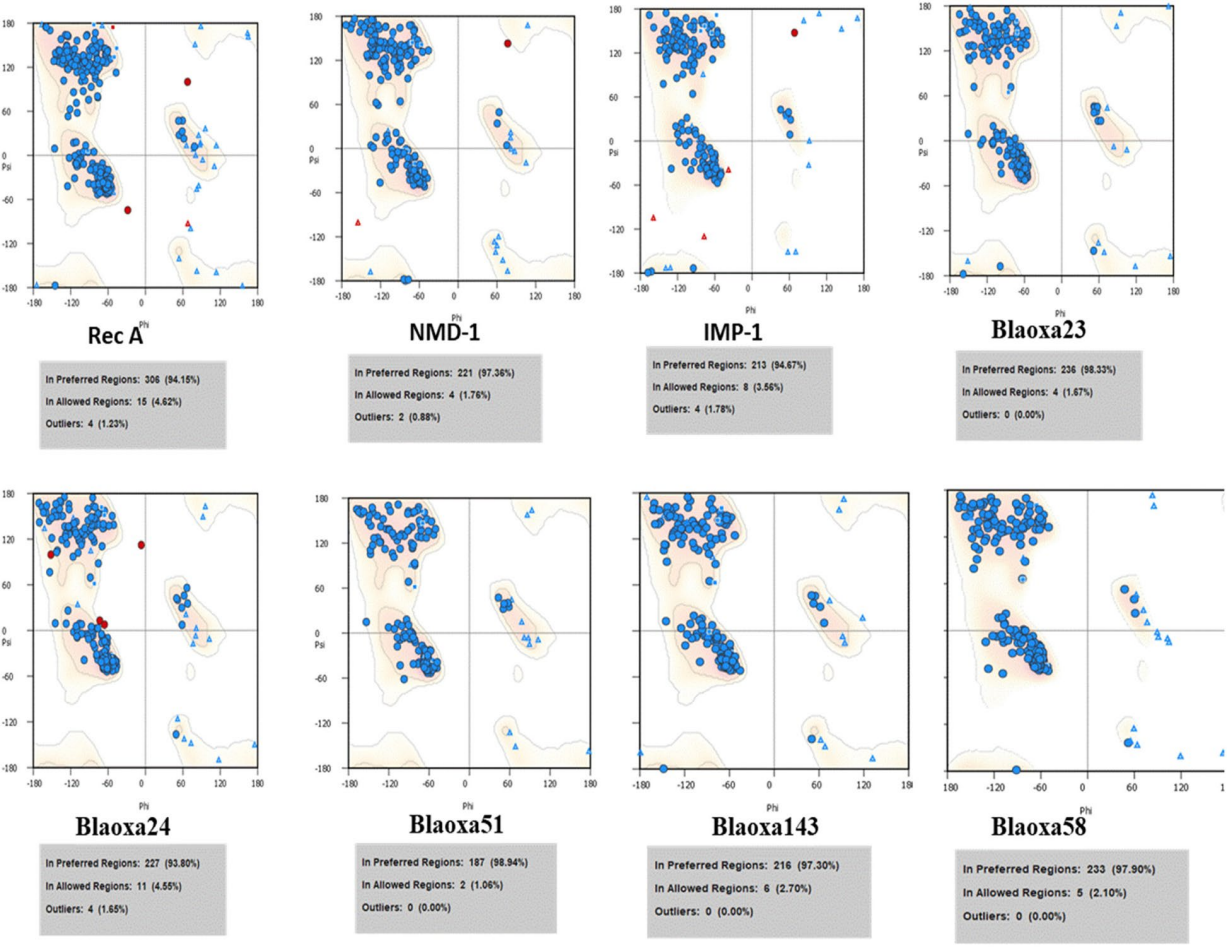
The Ramachandran graphs of all selected proteins are mentioned

IMP-1 consists of 246 amino acids. IMP-1 consists of 26% α-helices, 34% β-sheets, 38% coils and 21% turns, respectively. The Ramachandran plots and values of IMP-1 indicated that 94.67% of IMP-1 residues were present in preferred region and 3.56% residues were lying in the allowed region. Rec A is having 349 amino acids. The structural analysis revealed that rec-A is composed of 38% α-helices, 29% β-sheets, 31% coils and 12% turns. The Ramachandran plots and values of blaOXA-143 indicated that 94.15% of protein amino acids were present in preferred region and 4.62% residues were lie in allowed region (Fig. 1).

### Chemistry of carbapenem

**Fig. 2:**
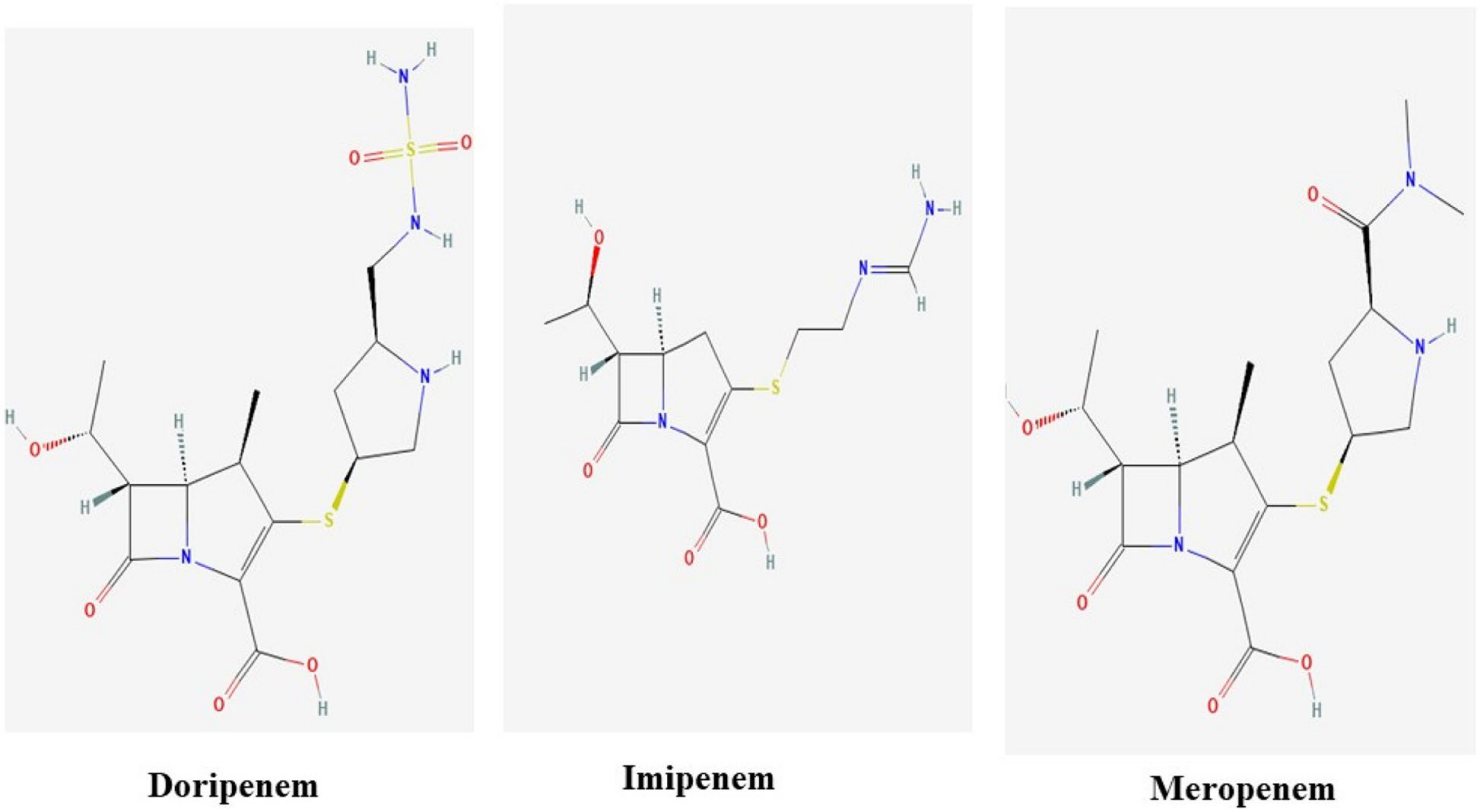
Two-dimensional (2-D) images of selected ligands

The Doripenem (C_15_H_24_N_4_O_6_S_2_) (PubChem ID 73,303) has a molecular weight of 420.5 g/mol. Doripenem is a broad-spectrum carbapenem antibiotic used primarily for the treatment of aerobic gram-negative bacterial infections (https://www.ncbi.nlm.nih.gov/books/n/livertox/Doripenem/). Imipenem (C_12_H_17_N_3_O_4_S) (PubChem ID 104,838), ’ is a broad-spectrum, intravenous beta-lactam antibiotic of the carbapenem subgroup. It has a role as an antibacterial drug. It is a beta-lactam antibiotic allergen and a member of carbapenems (http://www.ebi.ac.uk/chebi/searchId.do?chebiId=CHEBI:471744). Meropenem (C_12_H_17_N_3_O_4_S) (PubChem ID 441,130) is the anhydrous form of meropenem, a broad-spectrum carbapenem with antibacterial properties, synthetic Meropenem inhibits cell wall synthesis in gram-positive and gram-negative bacteria (Fig. 2). It penetrates cell walls and binds to penicillin-binding protein targets. Meropenem acts against aerobes and anaerobes (https://www.ncbi.nlm.nih.gov/books/n/livertox/Meropenem/).

### Active site prediction of selected proteins

**Fig. 3:**
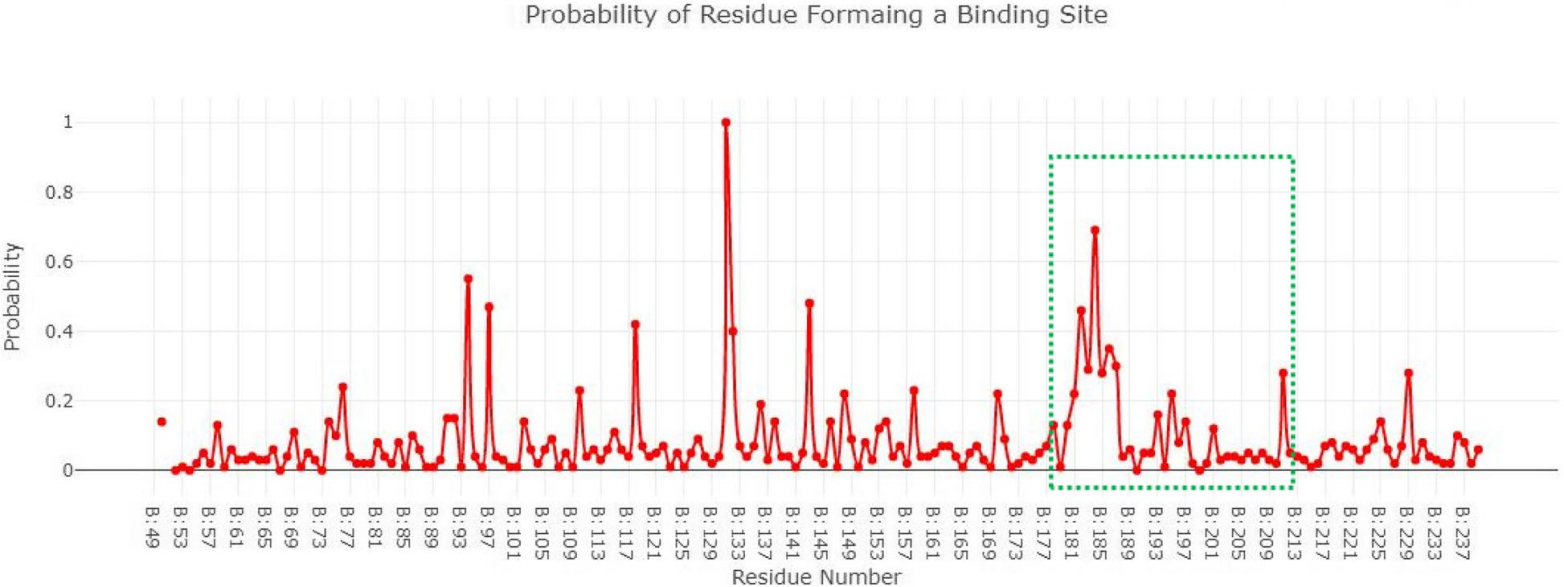
Structure of blaOXA-51 and probability of amino acids involved in the formation of the binding pocket

The active site is the functional part of proteins as it catalyzes the chemical reaction. Figure 3 showed the blaOXA-51 structure in a surface format the and probability of amino acids involved in the formation of the binding pocket. The Depth Residue results showed that the active site of blaOXA-51 contained 11 amino acids Lys182, Ser183, Gly184, Val193, Trp195, Val208, Ala209, Phe210, Ser211, Leu212, and Leu214 respectively.

### Active site of blaOXA-23

**Fig. 4:**
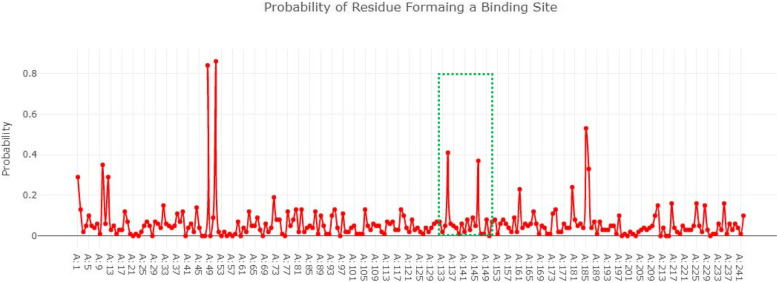
The binding pocket of blaOXA-23-like comprises 12 amino acids with the probability values in the range from 0 to 0.4

The binding pocket of blaOXA-23-like comprises 12 amino acids (Leu135, Glu146, Ser161, Try180, Lys185, Thr186). These binding pocket residues have good probability values in the range from 0 to 0.4 value as shown in Fig. 4.

### Active site of blaOXA-23

**Fig. 5:**
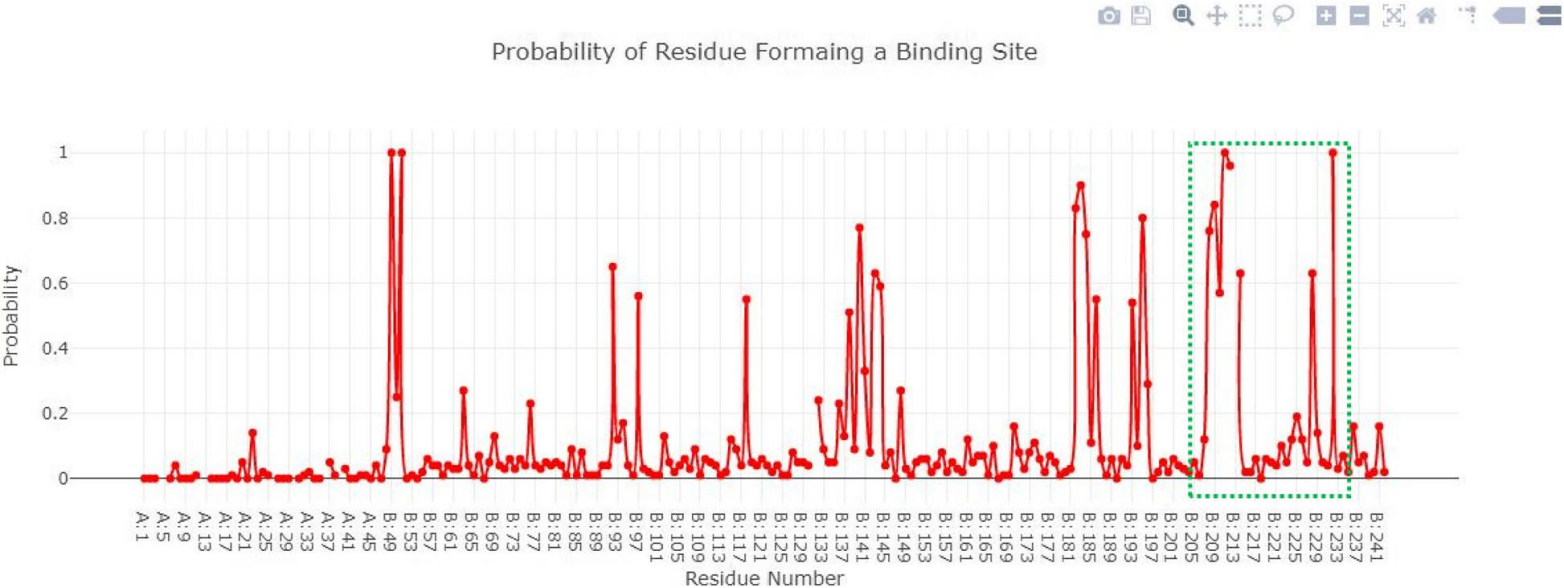
The binding pocket of blaOXA-23 consists of three amino acids and have a probability greater than 0 to 1

The binding pocket of blaOXA-23 consists of three amino acids (Leu212, Met214, Ser229). All the amino acids selected for the active site have a probability greater than 0 to 1 as shown in Fig. 5.

### Active site of IMP-1

**Fig. 6:**
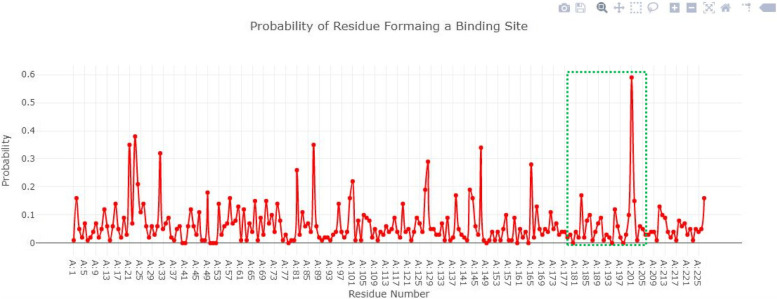
The IMP-1 structure in surface format and the probability of amino acids involved in the formation of the binding pocket

Figure 6 showed the IMP-1 structure in surface format and the probability of amino acids involved in the formation of the binding pocket. The Depth Residue results showed that the active site of IMP-1 contained 05 amino acids Lys165, Try167, Gly168, His201 and Ser202 respectively.

### Active site of rec A

**Fig. 7:**
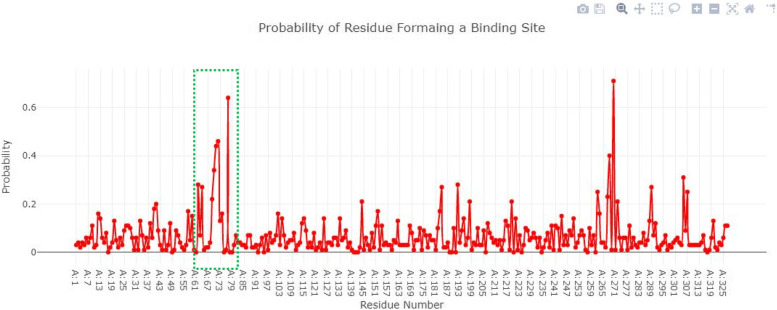
The binding pocket of rec A consists of seven amino acids and have probability greater than 0 to 1

The binding pocket of rec A consists of seven amino acids (Glu62, Try64, Ser69, Gly70, Lys71, Thr72, Gln77). All the amino acids selected for the active site have probability greater than 0 to 1 as shown in Fig. 7.

### Active site of blaOXA-143

**Fig. 8:**
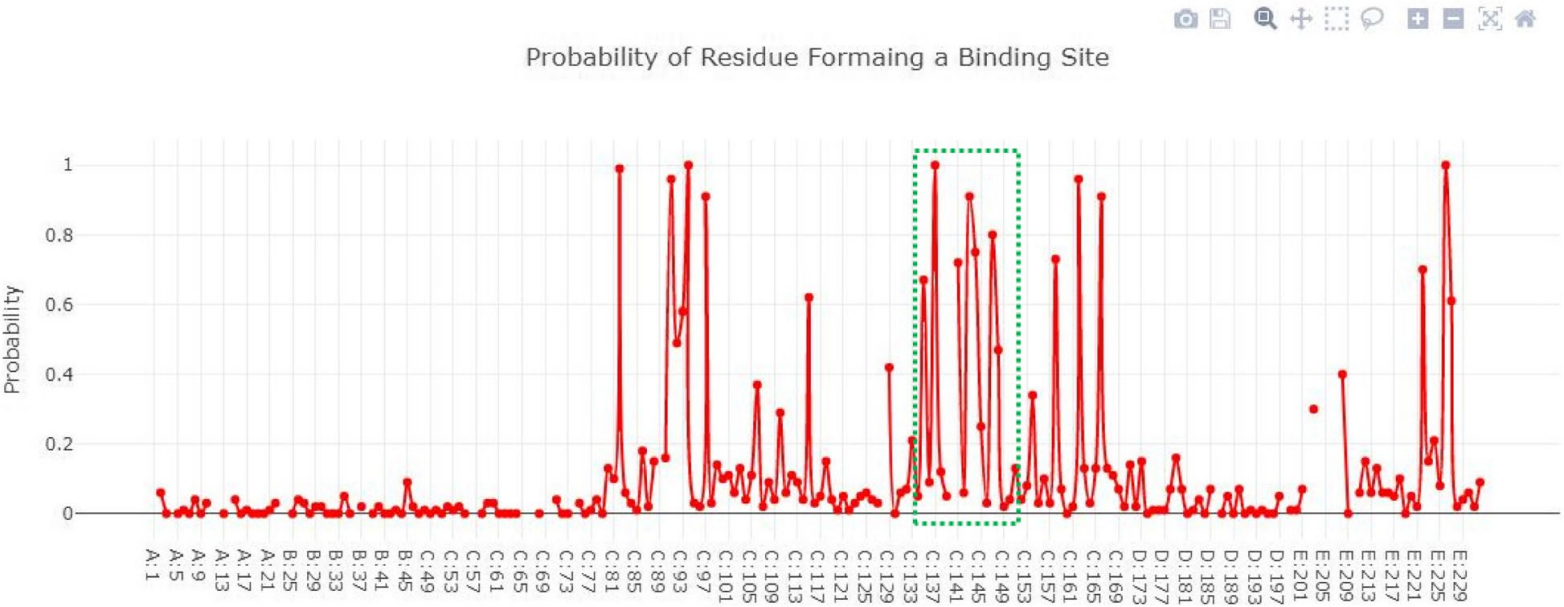
The binding pocket of blaOXA-143 consists of seven amino acids and have probability greater than 0 to 1

The binding pocket of blaOXA-143 consists of seven amino acids (Ile135, Pro137, Val141, Phe143, Ala144, Phe147, and Ala148). All the amino acids selected for active site have probability greater than 0 to 1 as shown in Fig. 8.

### Active site of blaOXA-24

**Fig. 9:**
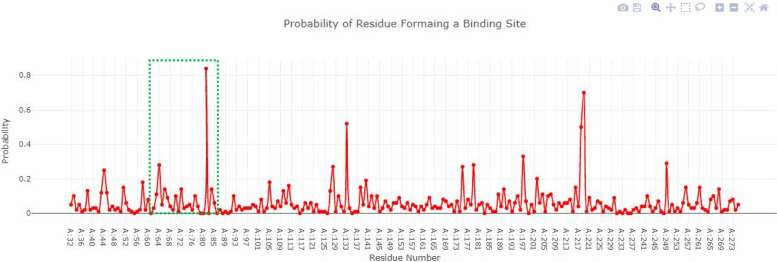
The binding pocket of blaOXA-24 consists of seven amino acids and have probability greater than 0 to 0.8

The binding pocket of blaOXA-24 consists of seven amino acids (Glu39, Leu63, Ser64, Thr65, Tyr66, Gly67, Leu70, Asn74). All the amino acids selected for active site have probability greater than 0 to 0.8 as shown in Fig. 9.

### Active site of NMD-1

**Fig. 10:**
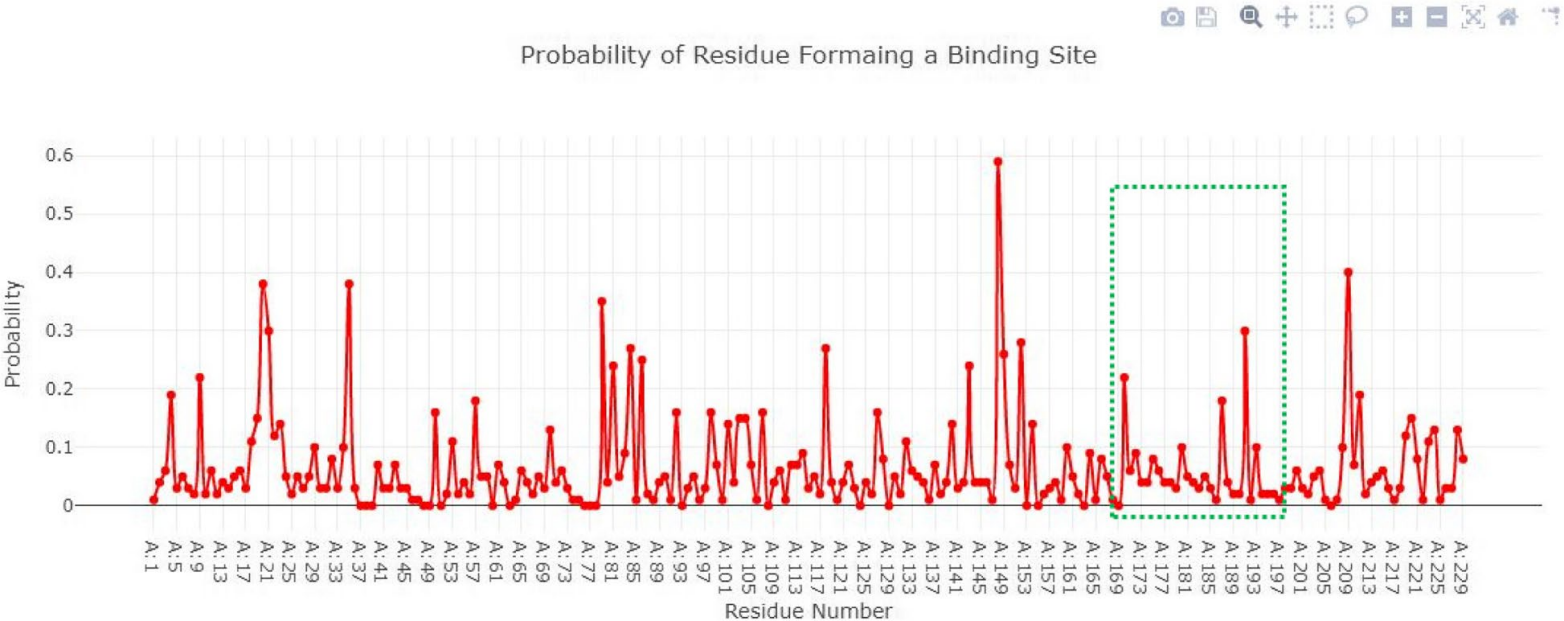
The binding pocket of NMD-1 consists of seven amino acids and probability greater than 0 to 0.3

The binding pocket of NMD-1 consists of seven amino acids (Lys140, Try143, His148, Thr149, Asn152, Thr154). All the amino acids selected for active site have probability greater than 0 to 0.3 as shown in Fig. 10.

### Binding pockets analysis and ligands interactions

**Fig. 11:**
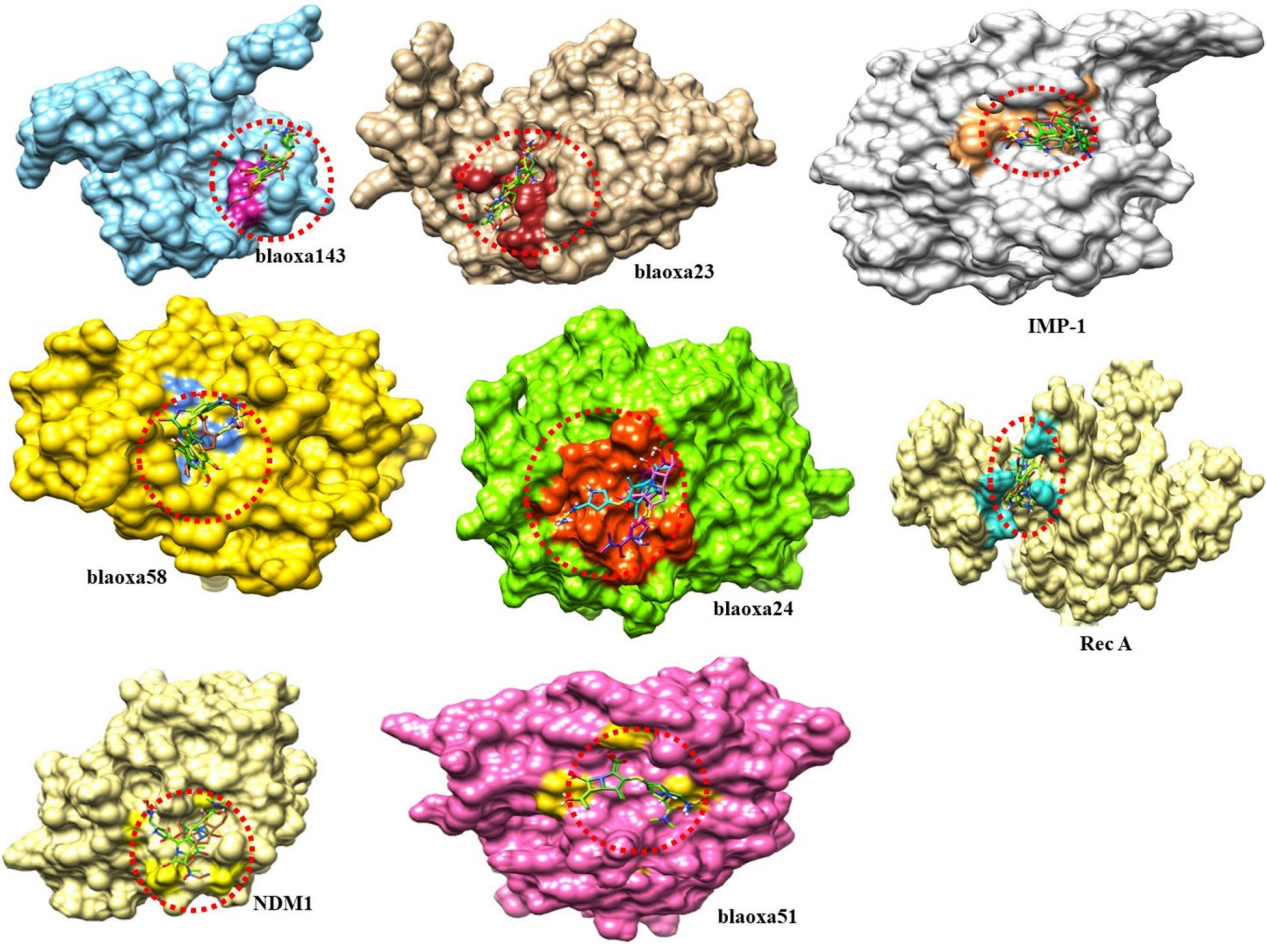
The docking results of all ligands in the active binding region of target proteins. The superimposition results of all three docking complexes showed that ligands bind in similar conformation and interactions pattern

The docking results showed that all ligands were confined in the active binding region of target proteins (blaOXA-51, blaOXA-23, blaOXA-58, blaOXA-24, blaOXA-143, NMD-1, IMP-1 and rec A). The superimposition results of all three docking complexes showed that ligands bind in similar conformational behavior and have similar binding interactions pattern as shown in the results in Fig. 11.

### Binding energies

The docking results of doripenem, imipenem and meropenem with blaOXA-23 demonstrated their binding energies (Table 1). The doripenem, imipenem and meropenem were docked to blaOXA-51 using PyRx. The top docking energy was -5.5 kcal/mol by imipenem and doripenem and meropenem showed a binding score of -5. 2 kcal/mol each. The top docking energy was -4.3 kcal/mol by imipenem and meropenem showed a binding score of -2.3 kcal/mol, while doripenem showcased a binding score of -3.4 kcal/mol. The doripenem, imipenem and meropenem were docked to blaOXA-58 using PyRx. The top docking energy was -8.8 kcal/mol by doripenem and meropenem while imipenem showed a binding score of -4.2 kcal/mol. The docking results of doripenem, imipenem and meropenem with IMP-1 demonstrated their binding energies. The top docking energy was -5.7 kcal/mol by meropenem and doripenem showed a binding score of -5.3 kcal/mol, while imipenem showed a binding score of -4.5 kcal/mol (Table 1).

**Table 1:** Docking energy of blaOXA-1, blaOXA-23, blaOXA-58, blaOXA-143, blaOXA-24, IMP-1, recA and NMD-1 against carbapenem antibiotics

**Proteins**	**Binding Energies (Kcal/mol)**		
	**Doripenem**	**Imipenem**	**Meropenem**
**blaOXA-51**	-5.2	-5.5	-5.2
**blaOXA-23**	-3.4	-4.3	-2.3
**blaOXA-58**	-4.9	-4.2	-4.9
**IMP-1**	-5.3	-4.5	-5.7
**rec A**	-3.8	-4.9	-3.6
**blaOXA-143**	-2.5	-3	-1.9
**blaOXA-24**	-5.5	-3.9	-4.4
**NMD-1**	-1.5	-4	-3.3

The doripenem, imipenem and meropenem were docked to Rec A using PyRx. The top docking energy was -4.9 kcal/mol by imipenem and meropenem showed binding energy of -3.6 kcal/mol each while doripenem showed a binding score of -3.9 kcal/mol. The docking results of doripenem, imipenem and meropenem with blaOXA-143 demonstrated their binding energies. The top docking energy was -3.0 kcal/mol by imipenem and meropenem showed a binding score of -1.9 kcal/mol, while doripenem showed a binding score of -2.5 kcal/mol.

Doripenem, imipenem, and meropenem docking findings with blaOXA-24 confirmed their binding energies. Doripenem had the highest docking energy of -5.5 kcal/mol, meropenem had a binding score of -4.0 kcal/mol, and imipenem had a binding score of -3.9 kcal/mol.

PyRx was used to dock the doripenem, imipenem, and meropenem to NMD-1. Docking energies for doripenem were all – 4.0 kcal/mol, whereas meropenem had a docking energy of -3.3 kcal/mol and imipenem was -1.50 kcal/mol. Selim et al. 2022 [21] reported that the docking energy of blaOXA-24, blaOXA-23, blaOXA-143 and blaOXA-51 are -10.39, -13.21, -10.29 and -8.52 kcal/mol respectively toward only imipenem.

### Hydrogen bonding analysis against blaOXA-51

Hydrogen and hydrophobic interactions are used to evaluate the bonding interactions of docking complexes. In the doripenem blaOXA-51 docking complex, five Hydrogen bonds have been observed at different residual positions within the active region of the target protein, Ala-209, Asn-213 Ser-232. The doripenem shares the two hydrogen bonds with Ala-209 having a distandistance08Å and2.13 Å, two hydrogen bonds with Asn-213 2.17 Å and1.92 Å respectively as shown in Table 2, and another hydrogen bond was observed between doripenem and blaOXA-51 with Ser-232 having a bond distance of 1.86 Å.In imipenem- blaOXA-51 docking results, a couple of hydrogen bonds were observed between imipenem and blaOXA-51 at the position of Tyr-118 and Glu-143 having bonding distances of 2.86 Å and 2.58 Å respectively, while meropenem formed a single hydrogen bond at Ser-232 with the bond distance of 2.20 Å (Table 2).

**Table 2:** The bond distance between amino acids and ligands viewed in the docking complex of blaOXA-51

**Ligands**	**Amino acids**	**Bond distance (**Å**)**	**Bond nature**
Doripenem	Ala-209	2.13	Hydrogen Bond
	Ala-209	2.08	Hydrogen Bond
	Asn-213	1.92	Hydrogen Bond
	Asn-213	2.17	Hydrogen Bond
	Ser-232	1.86	Hydrogen Bond
Imipenem	Tyr-113	2.86	Hydrogen Bond
	Glu-143	2.58	Hydrogen Bond
Meropenem	Ser-232	2.20	Hydrogen Bond

**Fig. 12:**
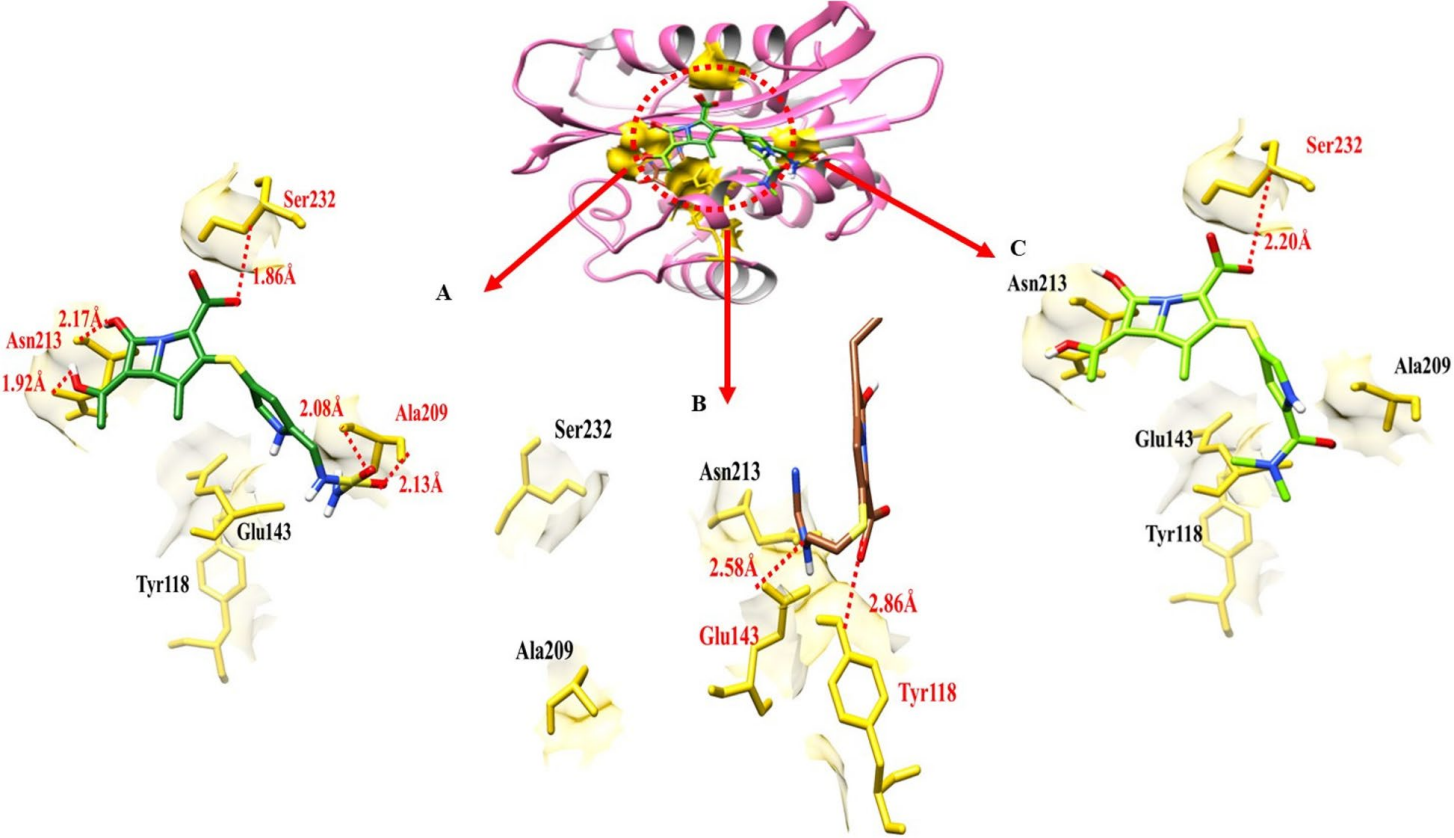
This figure showed binding interactions between the target protein (blaOXA-51) and three ligands

The target protein is indicated with the color orchid and grey in ribbon line format. The binding active amino acids all around ligands are outlined in yellow as shown in Fig. 12. Hydrogen bonds are also shown between blaOXA-51 amino acids and ligands at different distances, respectively. The red dotted lines indicate the hydrogen bond indicates the binding distance in angstrom (Å).

### Hydrogen bonding analysis against blaOXA-23

Doripenem- blaOXA-23 docking complex shows three hydrogen bonds in the active region of the target protein, at Ser-48, Lys-51, and Lys-185 having a bond distance of 1.69 Å, 2.98 Å and 2.11 Å respectively (Table 3). Meropenem established four hydrogen bonds at Lys-51, Met-92, Ala-96 and Asp-191. The bond distance calculated was 1.41 Å, 1.96 Å, 1.85 Å, and 2.35 Å respectively. imipenem- blaOXA-23 docking data shows that two hydrogen bonds were formed between imipenem and blaOXA-23 at Met-90 and Trp-188. The bond distance between imipenem and Met-90 was 1.79 Å while 2.97 Å with trp-188.

**Table 3:** The distance between amino acids and ligands in the docking complex of blaOXA-51

**Ligands**	**Amino acids**	**Bond distance (**Å**)**	**Bond nature**
Doripenem	Ser-48	1.69	Hydrogen Bond
	Lys-51	2.98	Hydrogen Bond
	Lys-185	2.11	Hydrogen Bond
Imipenem	Trp-188	2.97	Hydrogen Bond
	Met-190	1.79	Hydrogen Bond
Meropenem	Lys-51	1.41	Hydrogen Bond
	Met-92	1.96	Hydrogen Bond
	Ala-96	1.85	Hydrogen Bond
	Asp-191	2.35	Hydrogen Bond

**Fig. 13:**
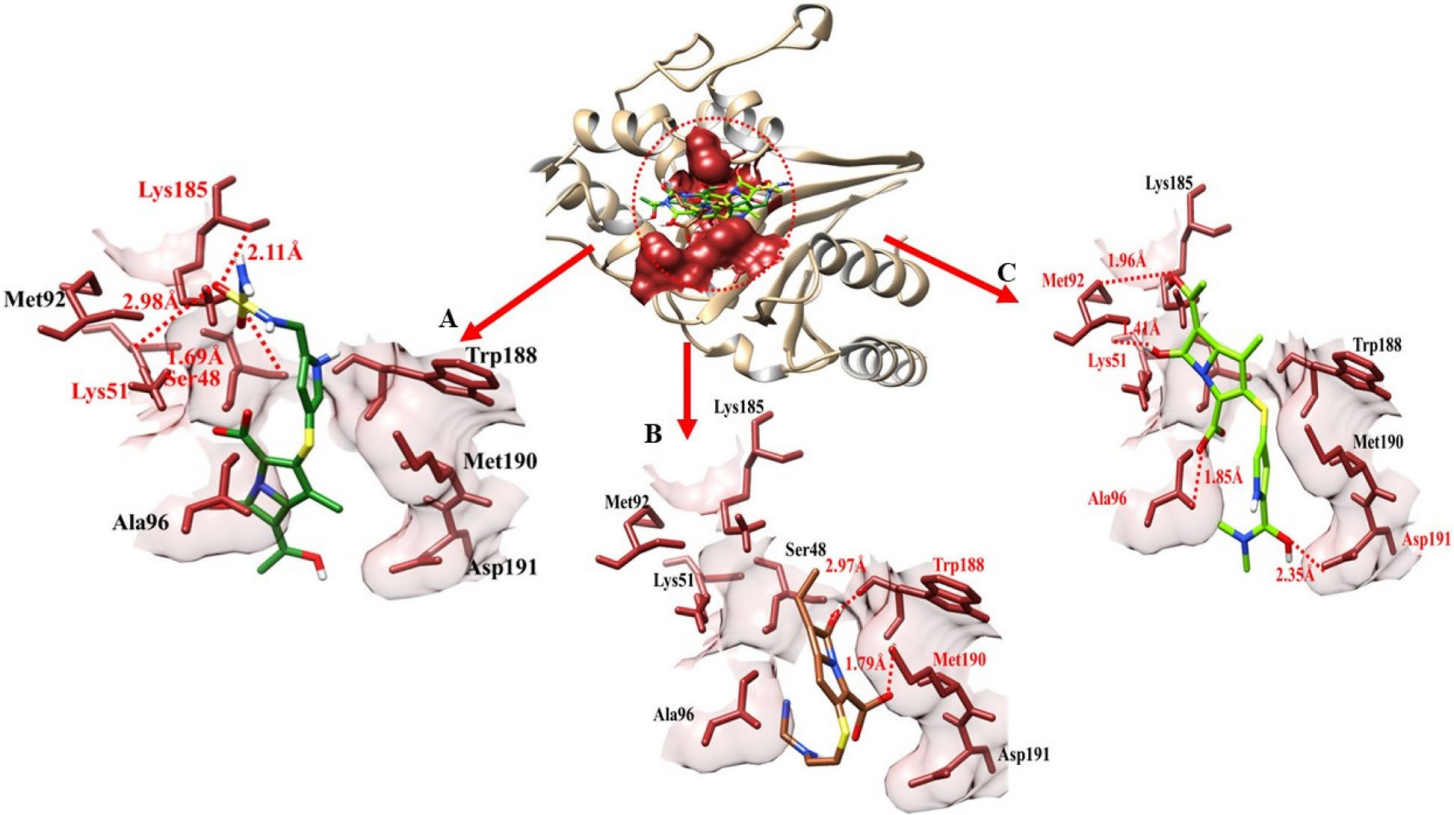
Target protein (blaOXA-51), three ligands, and their interactions

The target protein is indicated with the color dark khaki and light grey in ribbon line format. The binding active amino acids all around ligands are outlined in sienna color (Fig. 13). Hydrogen bonds are also shown between blaOXA-51 amino acids and ligands at different distances, respectively. The red dotted lines indicate the binding distance in angstrom (Å).

### Docking results with blaOXA-58

The doripenem- blaOXA-58 docking complex reveals four hydrogen bonds in the active region of the target protein, at Ala-209, Ala-211, and Ser-229, with bond distances of 3.06 Å, 2.26 Å, and 2.76 Å,2.38 Å correspondingly (Table 4). Meropenem formed two hydrogen bonds, one with Ala-209 and and Ser-229. It was found that the bond distances were 2.41 Å and 3.21 Å. There were two hydrogen bonds established between imipenem and blaOXA-58 at the Ala-211 having a distance of 2.11 Å. And 2.64 Å as shown in Table 4.

**Table 4:** The distance between amino acids and ligands in the docking complex of blaOXA-58

**Ligands**	**Amino acids**	**Bond distance (**Å**)**	**Bond nature**
Doripenem	**Ala_209**	3.06	Hydrogen Bond
	Ala-211	2.26	Hydrogen Bond
	Ser-229	2.76	Hydrogen Bond
Imipenem	Ala-211	2.64	Hydrogen Bond
	Ala-211	2.19	Hydrogen Bond
Meropenem	Ala-209	2.41	Hydrogen Bond
	Ser-229	3.21	Hydrogen Bond

**Fig. 14:**
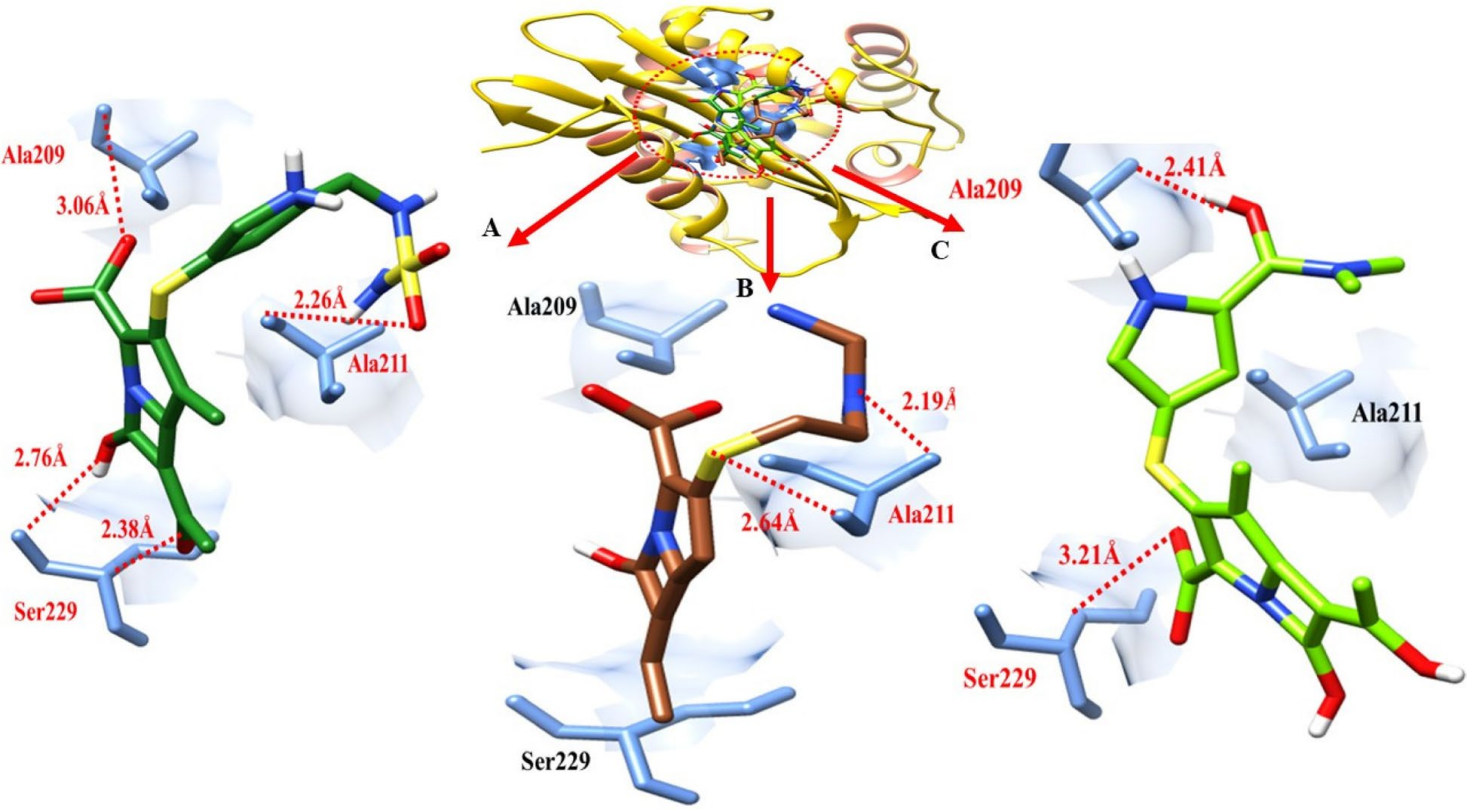
This figure shows the interactions of three ligands with the target protein (blaOXA-58)

The colours gold and sienna in ribbon line format identify the target protein. All around ligands, cornflower blue highlights delineate the binding active amino acids. blaOXA-58 amino acids and ligands are demonstrated to form hydrogen bonds at various distances. Red dots show the binding distance in angstroms (Fig. 14).

### IMP-1 binding analysis

Doripenem- IMP-1 docking complex shows three hydrogen bonds in the active region of the target protein, namely Gly-168, Ser-202and Glu-203 having bond distances 2.53 Å, 2.45 Å, 2.41 Å (Table 5). Imipenem shares two hydrogen bonds with His-201, and Ser-202 with bond distances of 2.27 Å and 2.87 Å. Meropenem established a single hydrogen bond at Lys-165 and a couple of hydrogen bonds at Ser-202 in the meropenem-IMP-1 docking data; the distance between meropenem and Lys-165 was 2.30 Å, compared to Ser-202 having bond distance of 2.70 Å and 2.73 Å, respectively as shown in Table 5.

**Table 5:** The distance between amino acids and ligands in the docking complex of IMP-1

**Ligands**	**Amino acids**	**Bond distance (**Å**)**	**Bond nature**
Doripenem	Gly-168	2.53	Hydrogen Bond
	Ser-202	2.45	Hydrogen Bond
	Glu-203	2.41	Hydrogen Bond
Imipenem	His-201	2.27	Hydrogen Bond
	Ser-202	2.87	Hydrogen Bond
Meropenem	Lys-165	2.30	Hydrogen Bond
	Ser-202	2.70	Hydrogen Bond
	Ser-202	2.73	Hydrogen Bond

**Fig. 15:**
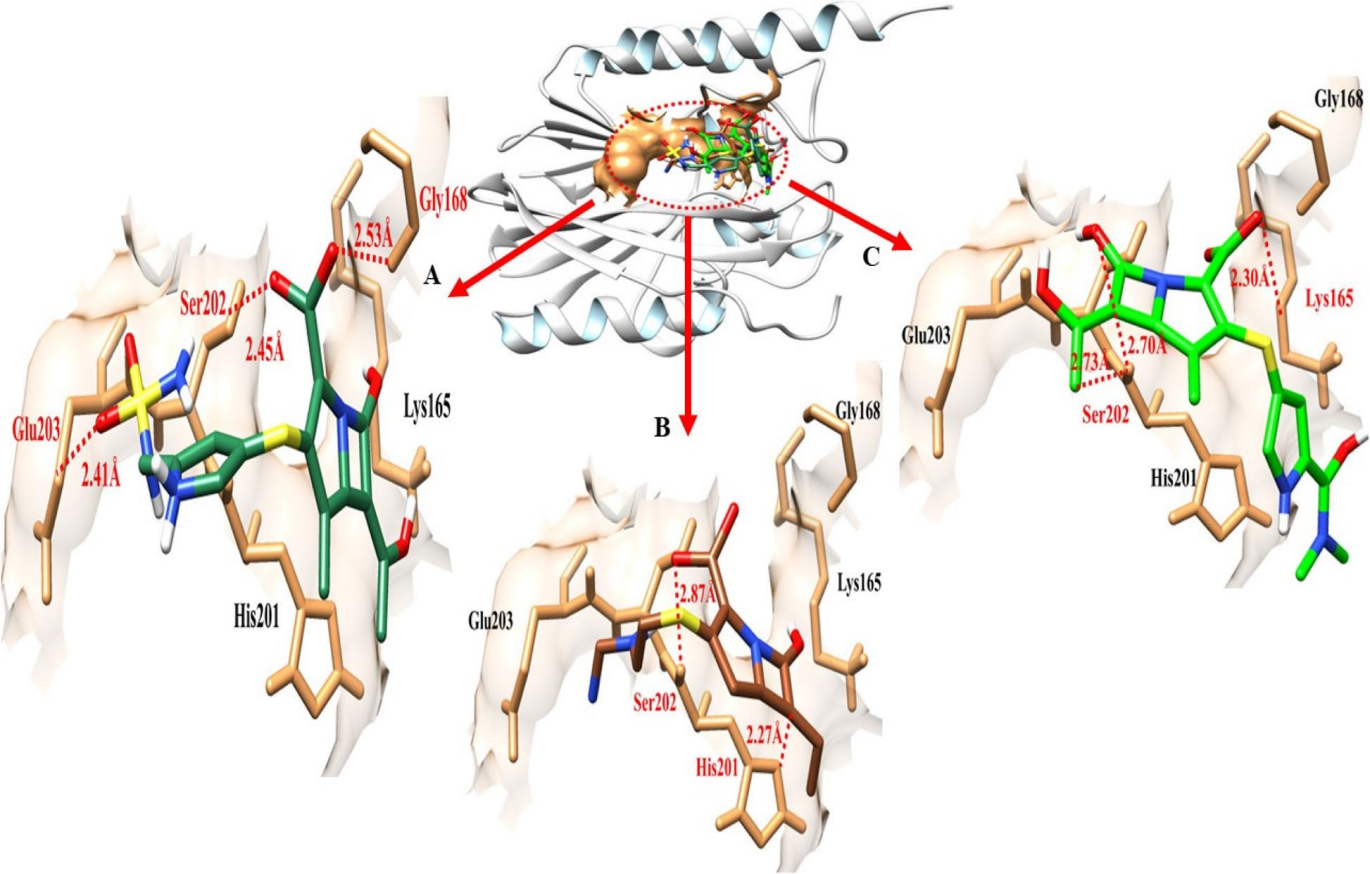
Target protein (IMP-1), three ligands, and their interactions

The target protein is indicated with the color grey and sky blue in ribbon line format. The binding active amino acids all around ligands are outlined in sandy brown color (Fig. 15). Hydrogen bonds are also shown between IMP-1 amino acids and ligands at different distances, respectively. The red dotted lines indicate the binding distance in angstrom (Å) as shown in Fig. 15.

### Rec A docking results

The doripenem- rec A docking complex exhibits three hydrogen bonds in the active region of the target protein, at Glu-232, Asn-268, and Arg-306 with a bond distance of 2.18 Å, 2.04 Å and 2.95 Å respectively. Imipenem bonded with rec A at Lys-265 and Asn-268. Bond distances were 2.38 Å and 2.93 Å respectively (Table 6). Meropenem and rec-A formed four hydrogen bonds at Asn-268, Arg-306, and Glu-309. A couple of bonds were formed at Asn-268 with bond distances of 2.40 Å and 2.85 Å. The bond distance between meropenem and Arg-306, and Glu-309 (rec A) was 2.57 Å and 2.67 Å respectively as shown in Table 6.

**Table 6:** The distance between amino acids and ligands in the docking complex of rec A

**Ligands**	**Amino acids**	**Bond distance (**Å**)**	**Bond nature**
Doripenem	Glu-232	2.18 Å	Hydrogen Bond
	Asn-268	2.04	Hydrogen Bond
	Arg-306	2.95	Hydrogen Bond
Imipenem	Lys-265	2.38	Hydrogen Bond
	Asn-268	2.93	Hydrogen Bond
Meropenem	Asn-268	2.40	Hydrogen Bond
	Asn-268	2.85	Hydrogen Bond
	Arg-306	2.57	Hydrogen Bond
	Glu-309	2.67	Hydrogen Bond

**Fig. 16:**
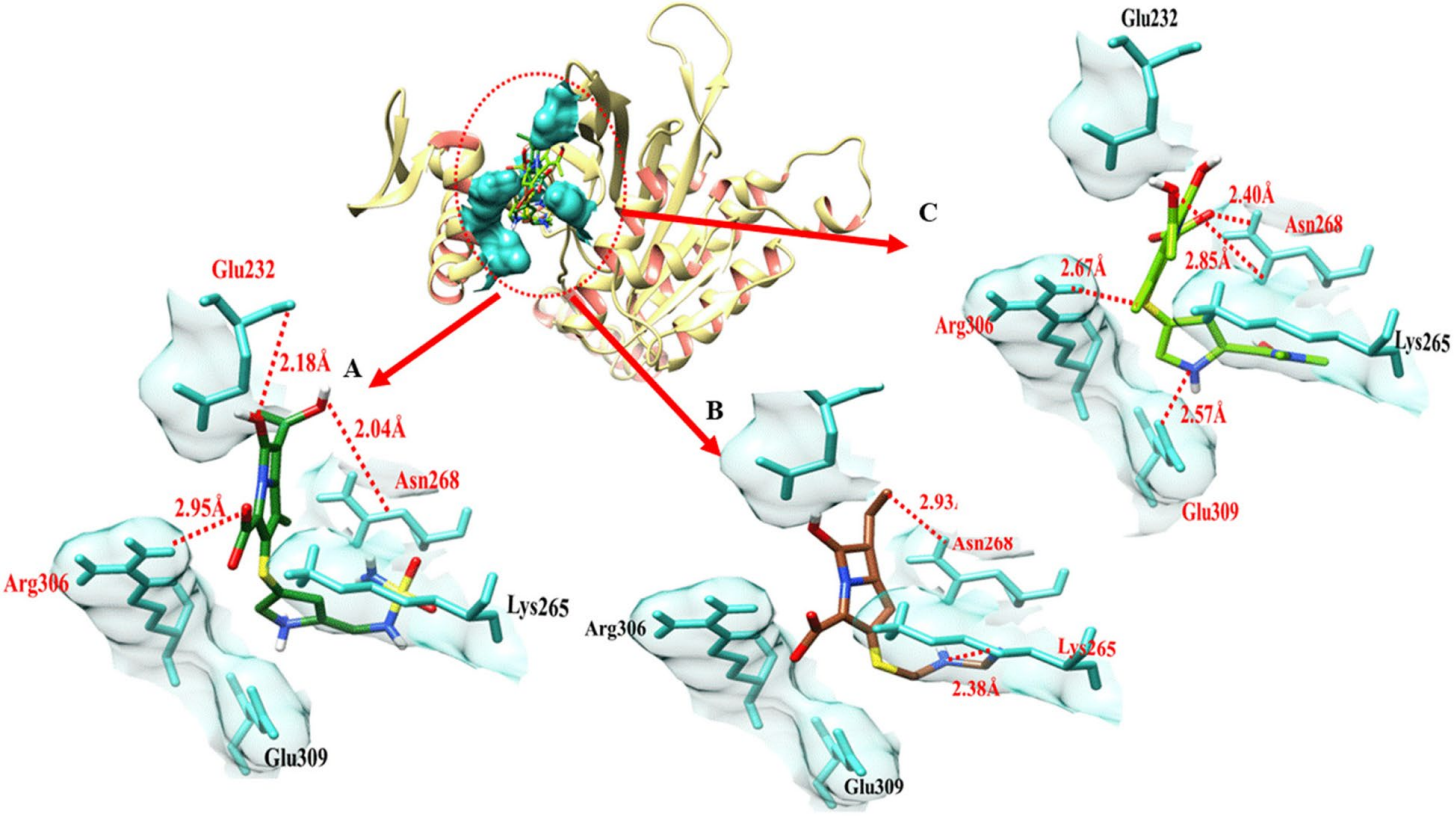
It shows *rec A*, three ligands, and their interactions

Gold and salmon ribbons identify the target protein. Skyblue binding amino acids surround ligands. IMP-1 amino acids and ligands have hydrogen bonding at varying distances. Red dots represent binding distance in angstrom Å Fig. 16.

### The blaOXA-143 binding analysis

In the doripenem blaOXA-143 docking complex, two hydrogen behave have been observed at different residual positions within the active region of the target protein. Couple of bonds at Glu-140 involved in hydrogen bond having a bond distance of 2.15 Å and 2.40 Å (Table 7). In imipenem- blaOXA-143 docking results, two hydrogen bonds were observed between imipenem and blaOXA-51 at the position of Ile-135 having bonding distances of 2.34 Å and 2.85 Å while meropenem formed two hydrogen bonds at Glu-140 with the bond distance of 2.93 Å and 3.18 Å (Table 7).

**Table 7:** The distance between amino acids and ligands in the docking complex of blaOXA-143is shown

**Ligands**	**Amino acids**	**Bond distance (**Å**)**	**Bond nature**
Doripenem	Glu-140	2.15	Hydrogen Bond
	Glu-140	2.40	Hydrogen Bond
Imipenem	Ile-135	2.34	Hydrogen Bond
	Asn-268	2.85	Hydrogen Bond
Meropenem	Glu-140	2.93	Hydrogen Bond
	Glu-140	3.18	Hydrogen Bond

**Fig. 17:**
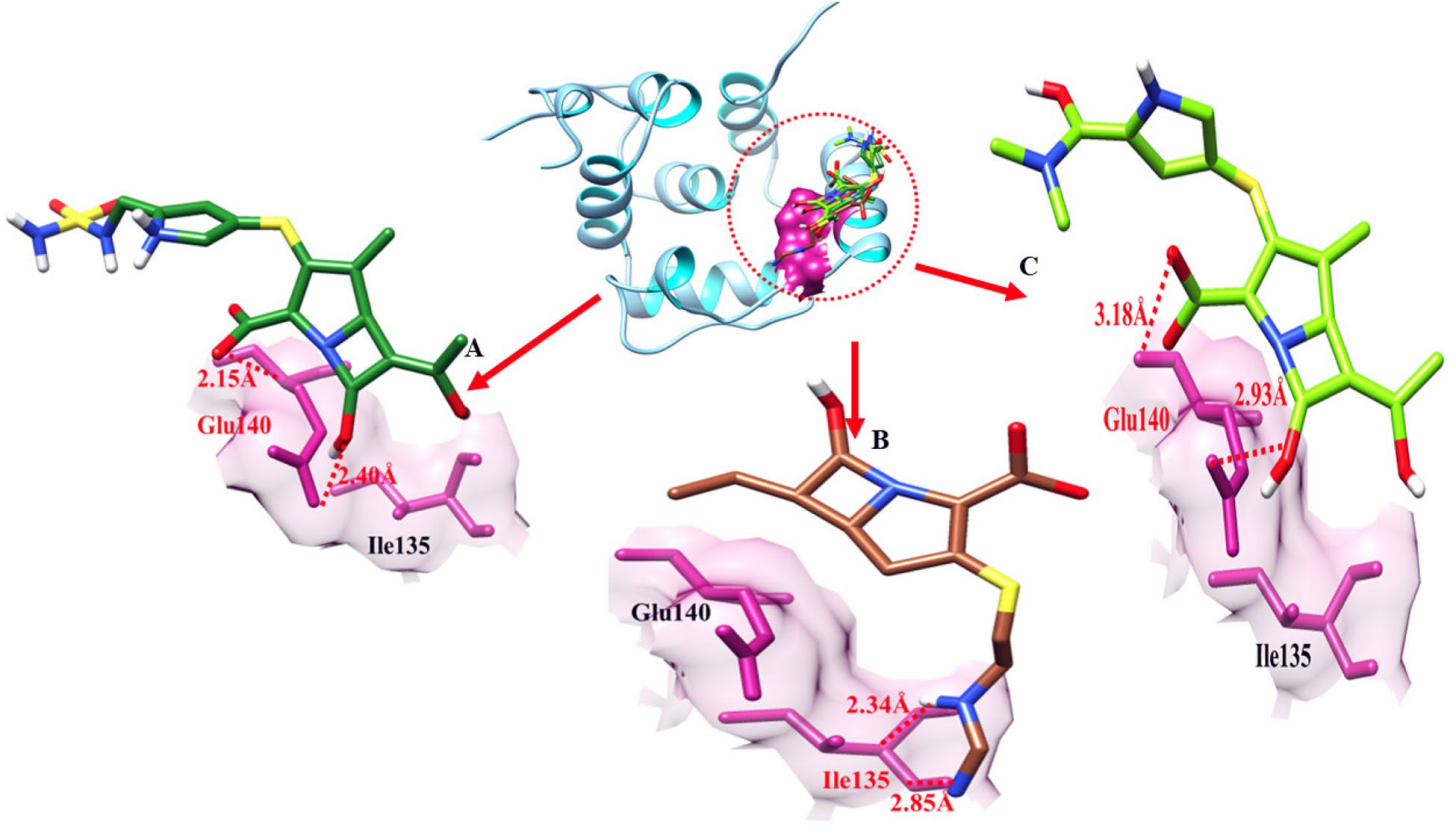
Target protein (blaOXA-143), three ligands, and their interactions

The target protein is indicated with the color dark cyan and cyan in ribbon line format. The binding active amino acids all around ligands are outlined in violet red color (Fig. 17). Hydrogen bonds are also shown between blaOXA-143 amino acids and ligands at different distances, respectively. The red dotted lines indicate the binding distance in angstrom (Å) as shown in Fig. 17.

### The blaOXA-24 binding analysis

Doripenem blaOXA-24 docking complex shows four hydrogen bonds in the active region of the target protein, namely Glu-39, Asn-62, Thr-65 and Gl-67 having bond distances 2.80 Å, 2.41 Å,2.20 Å and 2.58 Å respectively as shown in Table 8. Imipenem shares three hydrogen bonds with Asn-62, Thr-65 and Asn-74 with bond distances of 2.31 Å, 2.29 Å and 2.95 Å. Meropenem established a two-hydrogen bond at Asn-62 and Thr-65 in the meropenem- BlaOXA-24 docking data; the distance between meropenem and Asn-62 was 2.58 Å, compared to Thr-65 having bond distance of 2.40 Å (Table 8).

**Table 8:** The distance between amino acids and ligands in the docking complex of blaOXA-24

**Ligands**	**Amino acids**	**Bond distance (**Å**)**	**Bond nature**
Doripenem	Glu-39	2.80	Hydrogen Bond
	Asn-62	2.41	Hydrogen Bond
	Thr-65	2.20	Hydrogen Bond
	Gly-67	2.58	Hydrogen Bond
Imipenem	Asn-62	2.31	Hydrogen Bond
	Thr-65	2.29	Hydrogen Bond
	Asn-74	2.95	Hydrogen Bond
Meropenem	Asn-62	2.58	Hydrogen Bond
	Thr-65	2.40	Hydrogen Bond

**Fig. 18:**
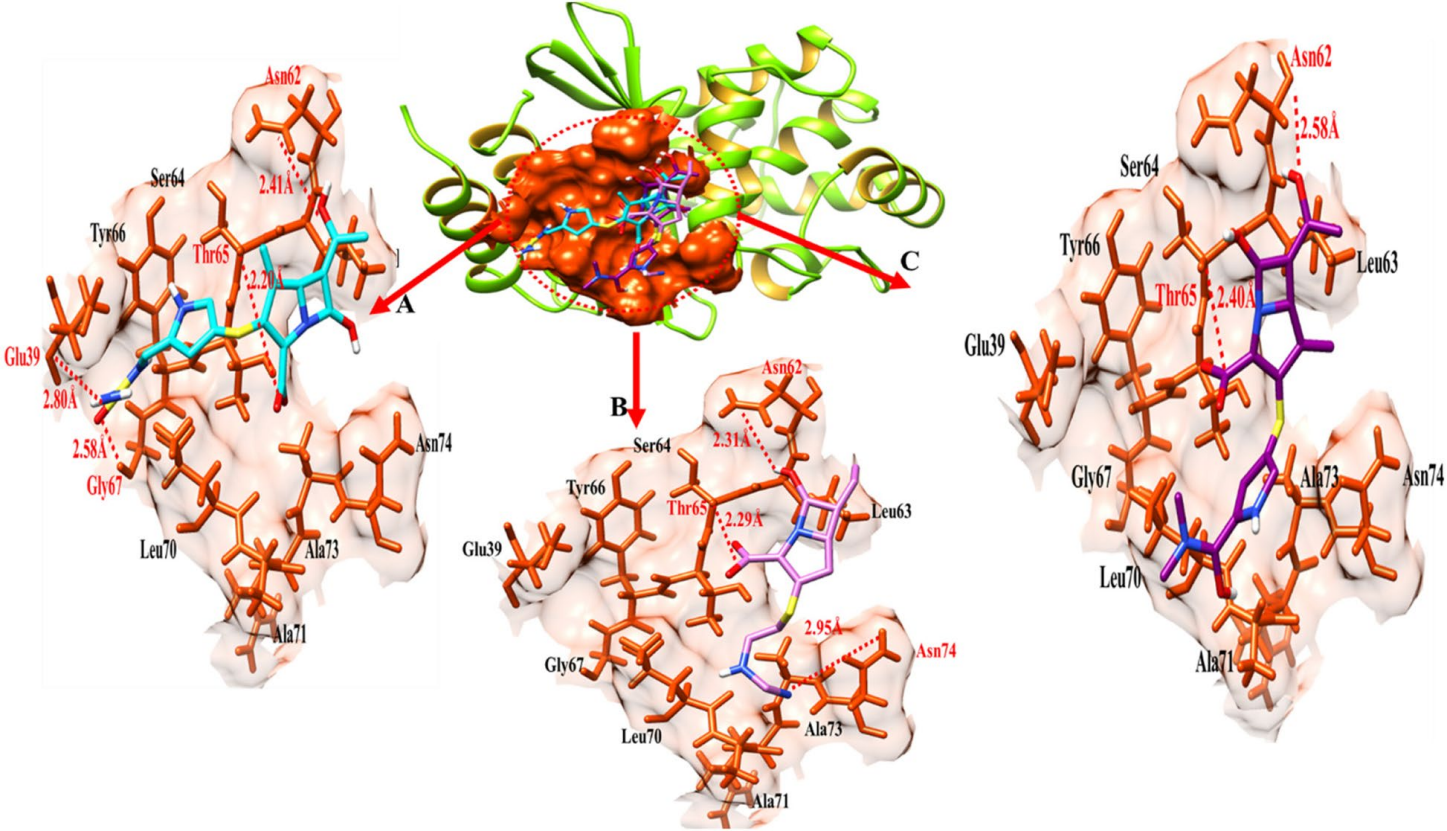
This figure shows binding interactions between the target protein (blaOXA-24) and three ligands

The target protein is indicated with the color and gold in ribbon line format. The binding active amino acids all around ligands are outlined in brown (Fig. 18). Hydrogen bonds are also shown between blaOXA-24 amino acids and ligands at different distances, respectively. The red dotted lines indicate the hydrogen bond indicates the binding distance in angstrom (Å) as shown in Fig. 18.

### Docking results with NMD-1

The Doripenem- NMD-1docking complex reveals two hydrogen bonds in the active region of the target protein, at Leu-139 and Ala-198, with bond distances of 2.25 Å, 2.36 Å correspondingly (Table 9). To begin with, Meropenem formed single hydrogen bonds with Asn-125. It was found that the bond distances were 2.00 Å. There were two hydrogen bonds established between the imipenem and NMD-1 at the Glu-129 and Leu-139 regions of the imipenem molecule. Glu-129 had a bond distance of 3.38 Å, whereas Leu-139 was 3.09 Å away (Table 9).

**Table 9:** The distance between amino acids and ligands in the docking complex of NMD-1

**Ligands**	**Amino acids**	**Bond distance (**Å**)**	**Bond nature**
Doripenem	Leu-139	2.25	Hydrogen Bond
	Ala-198	2.36	Hydrogen Bond
Imipenem	Glu-129	3.38	Hydrogen Bond
	Leu-139	3.09	Hydrogen Bond
Meropenem	Asn-125	2.00	Hydrogen Bond

**Fig. 19:**
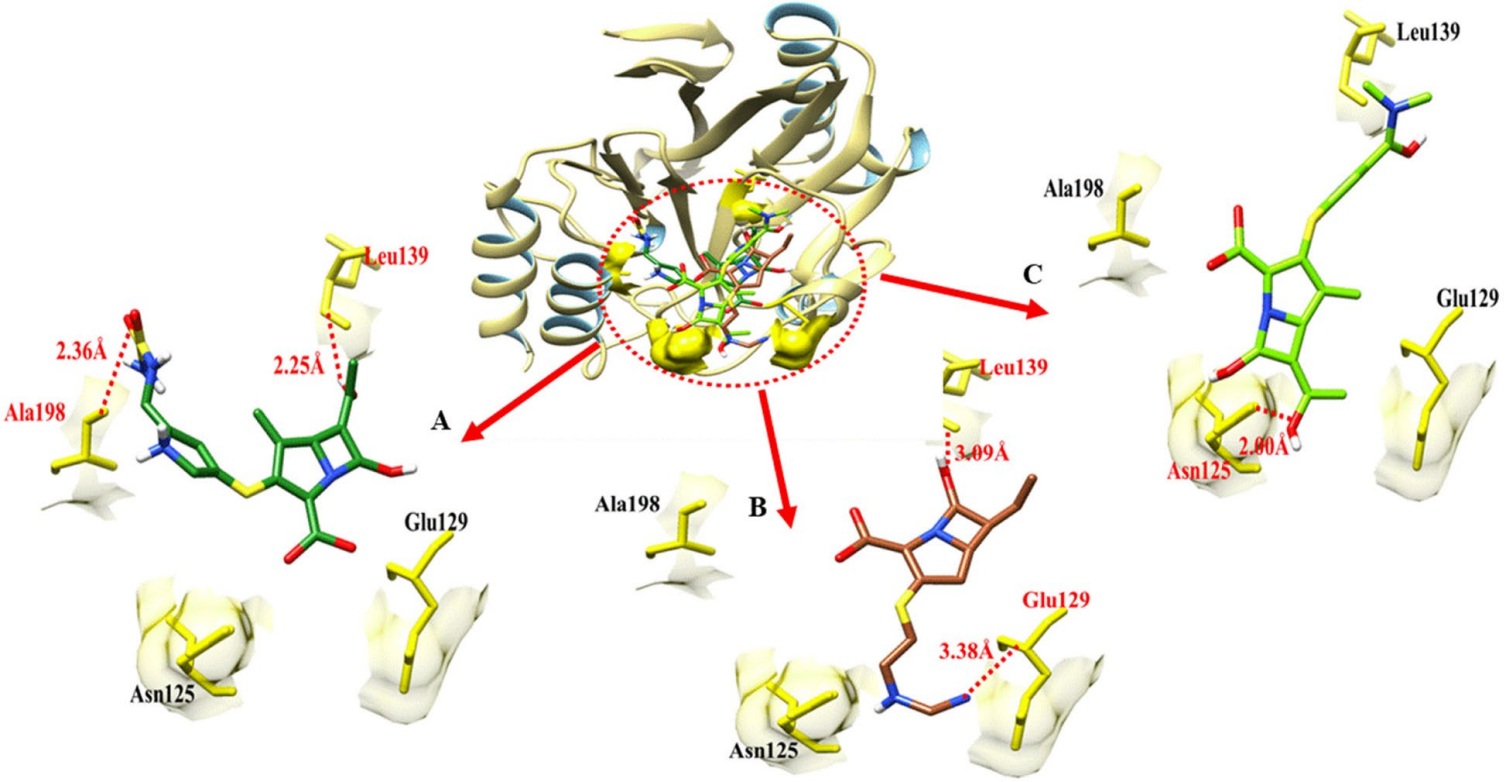
The figure showed binding interactions between the target protein (NMD-1) and three ligands

The target protein is indicated with the color khaki and sky blue in ribbon line format. The binding active amino acids all around ligands are outlined in yellow color as shown in Fig. 19. Hydrogen bonds are also shown between NMD-1 amino acids and ligands at different distances, respectively. The red dotted lines indicate the hydrogen bond and the binding distance in angstrom (Å) (Fig. 19).

## Conclusions

Our result may give a clear spot on carbapenem molecular docking data required for protein targeting the resistant gene need more advanced design of drugs and synthesis of new candidates to minimise the resistant crisis against *A. baumannii.*

## Data Availability

The datasets generated and/or analysed during the current study are available in the [Protein data bank (PDB) https://www.rcsb.org/] with the following accession numbers, 5KZH (blaOXA-51), 4JF5 (blaOXA-23), 4OH0 (blaOXA-58), 3G4P (blaOXA-24), 6NZ8 (blaOXA-143), and 6O3R (NMD-1). While IMP-1 and rec A were retrieved from the swiss model (https://swissmodel.expasy.org/interactive) with sequence entries Q6ZXZ6 (https://www.uniprot.org/uniprotkb/Q6ZXZ6) and PS50163 (https://prosite.expasy.org/PS50163) respectively.

## References

[1] World Health Organization (2017). Guidelines for the prevention and control of carbapenem-resistant Enterobacteriaceae, Acinetobacter baumannii and Pseudomonas aeruginosa in health care facilities.

[2] CDC A (2019). Antibiotic resistance threats in the United States.

[3] Murray CJ, Ikuta KS, Sharara F, Swetschinski L, Aguilar GR, Gray A, Han C, Bisignano C, Rao P, Wool E, Johnson SC (2022). Global burden of bacterial antimicrobial resistance in 2019: a systematic analysis. The Lancet.

[4] Zhang Y, Fan B, Luo Y, Tao Z, Nie Y, Wang Y, Ding F, Li Y, Gu D (2021). Comparative analysis of carbapenemases, RND family efflux pumps and biofilm formation potential among Acinetobacter baumannii strains with different carbapenem susceptibility. BMC Infect Dis.

[5] Wareth G, Brandt C, Sprague LD, Neubauer H, Pletz MW (2021). WGS based analysis of acquired antimicrobial resistance in human and non-human Acinetobacter baumannii isolates from a German perspective. BMC Microbiol.

[6] Yang MR, Wu YW (2022). Enhancing predictions of antimicrobial resistance of pathogens by expanding the potential resistance gene repertoire using a pan-genome-based feature selection approach. BMC Bioinformatics.

[7] Almihyawi RA, Naman ZT, Al-Hasani HM, Muhseen ZT, Zhang S, Chen G (2022). Integrated computer-aided drug design and biophysical simulation approaches to determine natural anti-bacterial compounds for Acinetobacter baumannii. Sci Rep.

[8] Abishad P, Niveditha P, Unni V, Vergis J, Kurkure NV, Chaudhari S, Rawool DB, Barbuddhe SB (2021). In silico molecular docking and in vitro antimicrobial efficacy of phytochemicals against multi-drug-resistant enteroaggregative Escherichia coli and non-typhoidal Salmonella spp. Gut pathogens.

[9] Chopjitt P, Wongsurawat T, Jenjaroenpun P, Boueroy P, Hatrongjit R, Kerdsin A (2020). Complete genome sequences of four extensively drug-resistant Acinetobacter baumannii isolates from Thailand. Microbiol Resour Announc.

[10] Pormohammad A, Mehdinejadiani K, Gholizadeh P, Nasiri MJ, Mohtavinejad N, Dadashi M, Karimaei S, Safari H, Azimi T (2020). Global prevalence of colistin resistance in clinical isolates of Acinetobacter baumannii: A systematic review and meta-analysis. Microb Pathog.

[11] Dent L, Ismail N, Robinson S, Rogers G, Pratap S, Marshall D (2011). Next-gen sequencing of multi-drug resistant Acinetobacter baumanii at Nashville General Hospital at Meharry. BMC Bioinformatics.

[12] Theriault N, Tillotson G, Sandrock CE (2021). Global travel and Gram-negative bacterial resistance; implications on clinical management. Expert Rev Anti Infect Ther.

[13] Zeshan B, Karobari MI, Afzal N, Siddiq A, Basha S, Basheer SN, Peeran SW, Mustafa M, Daud NH, Ahmed N, Yean CY (2021). The usage of antibiotics by COVID-19 patients with comorbidities: the risk of increased antimicrobial resistance. Antibiotics.

[14] Ten KE, Md Zoqratt MZ, Ayub Q, Tan HS (2021). Characterization of multidrug-resistant Acinetobacter baumannii strain ATCC BAA1605 using whole-genome sequencing. BMC Res Notes.

[15] Pettersen EF, Goddard TD, Huang CC, Couch GS, Greenblatt DM, Meng EC, Ferrin TE (2004). UCSF Chimera—a visualization system for exploratory research and analysis. J Comput Chem.

[16] DeLano WL (2002). Pymol: An open-source molecular graphics tool. CCP4 Newsl. Protein Crystallogr.

[17] Gopalakrishnan K, Sowmiya G, Sheik SS, Sekar K (2007). Ramachandran plot on the web (2.0). Protein Pept lett.

[18] Dallakyan S, Olson AJ (2015). Small-molecule library screening by docking with PyRx. Chemical Biology.

[19] Zahra N, Zeshan B, Qadri M M A, Ishaq M, Afzal M, et al. Phenotypic and Genotypic Evaluation of Antibiotic Resistance of Acinetobacter baumannii Bacteria Isolated from Surgical Intensive Care Unit Patients in Pakistan. Jundishapur J Microbiol. 2021;14(4):e113008. 10.5812/jjm.113008.

[20] Woon JJ, Teh CS, Chong CW, Abdul Jabar K, Ponnampalavanar S, Idris N (2021). Molecular Characterization of Carbapenem-Resistant Acinetobacter baumannii Isolated from the Intensive Care Unit in a Tertiary Teaching Hospital in Malaysia. Antibiotics.

[21] Selim S, Faried OA, Almuhayawi MS, Mohammed OA, Saleh FM, Warrad M (2022). Dynamic Gene Clusters Mediating Carbapenem-Resistant Acinetobacter baumannii Clinical Isolates. Antibiotics.

